# A Multicity Analysis of the Short-Term Effects of Air Pollution on the Chronic Obstructive Pulmonary Disease Hospital Admissions in Shandong, China

**DOI:** 10.3390/ijerph15040774

**Published:** 2018-04-17

**Authors:** Yi Liu, Jingjie Sun, Yannong Gou, Xiubin Sun, Xiujun Li, Zhongshang Yuan, Lizhi Kong, Fuzhong Xue

**Affiliations:** 1 Department of Epidemiology and Biostatistics, School of Public Health, Shandong University, 44, Wenhuaxi Street, Jinan 250012, China; liuyi238@sdu.edu.cn (Y.L.); sunxiubin@sdu.edu.cn (X.S.); xjli@sdu.edu.cn (X.L.); yuanzhongshang@sdu.edu.cn (Z.Y.); 2 Health and Family Planning Information Center of Shandong Province, 75, Yuhan Street, Jinan 250002, China; sunjingjie163@163.com (J.S.); gouyannong@163.com (Y.G.); 3 Shandong Academy of Environmental Science, 50, Lishan Street, Jinan 250013, China; sdhky2@163.com

**Keywords:** air pollution, chronic obstructive pulmonary disease, hospital admission, multicity analysis, generalized additive model

## Abstract

Although there is growing evidence linking chronic obstructive pulmonary disease (COPD) hospital admissions to the exposure to ambient air pollution, the effect can vary depending on the local geography, pollution type, and pollution level. The number of large-scale multicity studies remains limited in China. This study aims to assess the short-term effects of ambient air pollution (PM_2.5_, PM_10_, SO_2_, NO_2_) on chronic obstructive pulmonary disease hospital admissions from 2015 to 2016, with a total of 216,159 records collected from 207 hospitals in 17 cities all over the Shandong province, east China. Generalized additive models and penalized splines were applied to study the data whilst controlling for confounding meteorological factors and long-term trends. The air pollution was analyzed with 0–6 day lag effects and the percentage change of hospital admissions was assessed for a 10-μg/m^3^ increase in the air pollution levels. We also examined the percentage changes for different age groups and gender, respectively. The results showed that air pollution was significantly associated with adverse health outcomes and stronger effects were observed for females. The air pollution health effects were also impacted by geographical factors such that the air pollution had weaker health effects in coastal cities.

## 1. Introduction

Many studies across the world have shown that poor air quality adversely impacts people’s health [1,2,3,4,5]. The Global Burden of Disease assessments indicated that ca. 7 million deaths per year and 3.1% of the global disease burden could be attributed to ambient particulate matter pollution, placing it among the top health risk factors globally [6,7]. Exposure to high levels of air pollutant concentration is particularly associated with respiratory and cardiovascular problems [8,9,10,11,12]. In respiratory diseases, Chronic Obstructive Pulmonary Disease (COPD) is presently the fourth leading cause of death and it is predicted to become the third leading cause by 2030, making this disease one of the major health challenges in the future [13,14]. COPD led to ca. 3 million deaths in 2004, 90% of which were from developing countries [15].

Due to the fast development in industrialization and urbanization with a marked incline in oil and coal burning, poor air quality has become a severe problem in China [16,17,18]. Heavy smog caused by a combination of industrial pollution sources and adverse weather conditions has been appearing more and more often in northern China. Considering the very large number of people who may be exposed to substantial amounts of air pollution, this is an important and urgent health issue [19,20,21,22].

Compared with extreme conditions such as mortality or morbidity, hospital admission is more directly related to air pollution exposure. Most of the existing studies were conducted in developed countries such as Europe and the United States [23,24,25,26,27,28,29,30,31]. The number of studies concentrating on the relationship between air pollution levels in multicities and the corresponding hospital admissions in China is limited. There are a few studies focused only on a single city [32,33,34,35,36].

COPD, as a type of respiratory disease, can be exacerbated by air pollution. In this study, we aimed to assess the short-term association between air pollution and chronic obstructive pulmonary disease (COPD) hospital admissions collected from all the tertiary-level and secondary-level hospitals in the 17 cities of the Shandong province. We then explored the influence of different geography, air pollution types, and pollution levels on human health; hence, providing public health evidence for preventative measures and health management for different ambient pollution environments. Shandong is located in east China; the eastern part of the province is by the sea and the western and middle parts are hilly areas. In addition, Shandong is one of the most prosperous and heavily polluted provinces in the country, with significant coal mining, oil refineries, and metallurgical and mechanical sectors.

## 2. Materials and Methods

### 2.1. Hospital Admissions

Hospital admission data were obtained from the Health and Family Planning Information Center of the Shandong Province. Daily counts of COPD hospital admissions between 1 January 2015 and 31 December 2016 were constructed from individual records of all tertiary-level and secondary-level hospitals in Shandong; there is at least one tertiary-level hospital in each city. Each record contains the hospital name, date of service, age, gender, date of birth, current address, and disease ICD code (International Classification of Diseases, Tenth Revision (ICD-10) codes). Chronic obstructive pulmonary disease outcomes were considered based on the ICD-10 codes J40–44.

### 2.2. Air Pollution and Meteorological Data

**Figure 1:**
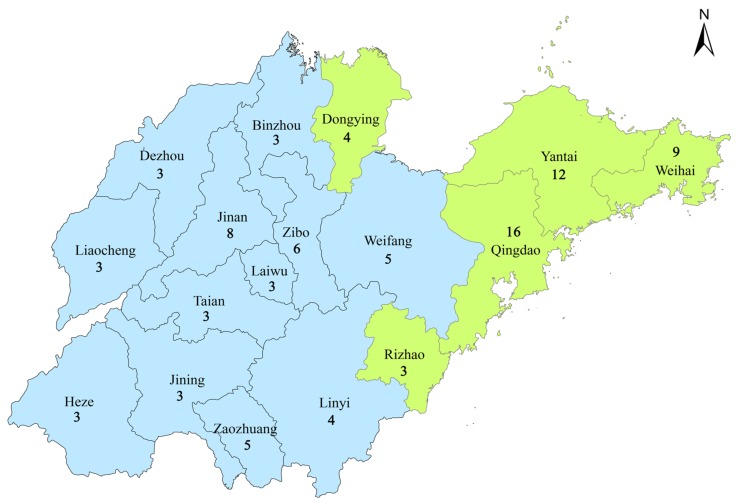
The locations of the 17 cities in the Shandong Province; the coastal cities are shown in the green color. The numbers represent the count of air quality monitoring stations in each city.

There are 93 air quality monitoring stations established all over Shandong by the China National Environmental Protection Administration. The number of air quality monitoring sites in each city are shown in Figure 1. Each air quality monitoring station provides hourly readings of PM_10_, PM_2.5_, SO_2_, and NO_2_ levels. Since the hospital admissions data were available daily, we aggregated the pollution data to daily counts by taking the mean value of all 24 hourly collected air pollution concentrations on each day. The meteorological data were obtained from the China Meteorological Administration Data Center (URL https://www.data.cma.cn/). These data were collected daily and included the location and altitude of the station, the daily mean, and the highest and lowest value of the monitored objects. In our study, the daily mean temperature, the relative humidity, and the wind speed were used to eliminate the confounding effects of weather.

### 2.3. Method of Analysis

We applied generalized additive Quasi-Poisson models to examine the associations between air pollution (PM_2.5_, PM_10_, SO_2_, NO_2_) and day-to-day variations in the COPD hospital admissions for the total patients, for males, for females, for patients aged over 65, and for patients under 65. The Quasi-Poisson distribution was adopted in order to overcome the overdispersion of hospital admissions data. Generalized additive models allow for highly flexible fitting as the outcome is assumed to be dependent on a sum of the smoothed and linear functions of the predictor variables. A smoothed function captures the nonlinear relationship between daily hospital admissions and the time-varying covariates such as the temperature and the calendar time [37,38]. The nonlinear term in generalized additive models can be estimated by using smoothing splines, which transform a possible nonlinear relationship into a linear form by separating the data into subintervals based on the basis functions. The smoothness of the splines depends on the number of basis functions: too many subintervals may lead to oversmoothness, whilst an inadequate number of subintervals leads to a rough model fitting. A penalized spline uses an overly large number of basis functions and penalizes excess curvature by using a penalty term. The trade-off between model fitting and model smoothness is controlled by the smoothing parameter which may be chosen by using a data-driven criterion such as generalized cross-validation (GCV) [39,40].

For each of the 17 cities in Shandong, the daily ambient air pollution (PM_10_, PM_2.5_, SO_2_, and NO_2_) concentrations for the 0–6 day lags were analyzed separately in association with daily hospitalizations. Briefly, we fitted the data into the following model:

(1) $$Log[E(Y_{i})]=\alpha +\beta X_{i}+ps(time)+\sum_{j}ps(Z_{ij})+DOW$$

where $E(Y_{i})$ is the expected number of daily COPD hospital admissions at day i, α is the intercept term, β is the regression coefficient, and $X_{i}$ is the pollutant measurements on day i. ps(time) denotes the penalized splines of calendar time and $ps(Z_{ij})$ denotes the penalized splines of the meteorological variables $Z_{j}$ such as the temperature, relative humidity, and wind speed, respectively. The dummy variable DOW is the indicator for the day of the week. All statistical analyses were performed with R V.3.4.3 (URL http://www.R-project.org) using the package mgcv (V.1.8–17). Then all results were presented as the percentage change in the relative risk (RR) of hospitalizations and its 95% confidence interval (CI) in association with a 10 μg/m^3^ increase in daily air pollutants.

## 3. Results

The locations of the 17 cities in Shandong Province are shown in Figure 1. There are 5 coastal cities: Dongying, Yantai, Weihai, Qingdao, and Rizhao. In this study, the total number of COPD hospital admissions during the study period was 216,159, the numbers of male and female records were 131,499 and 84,660, respectively. There were 162,601 records for patients over 65 years old, and 53,558 records for those under 65. The largest number of total hospital admissions during the two-year study time was 26,653 in the city of Weifang, while the smallest number was 4026 from the city of Dongying. The details of the hospital admissions in the 17 cities in Shandong can be found in Table 1.

**Figure 2:**
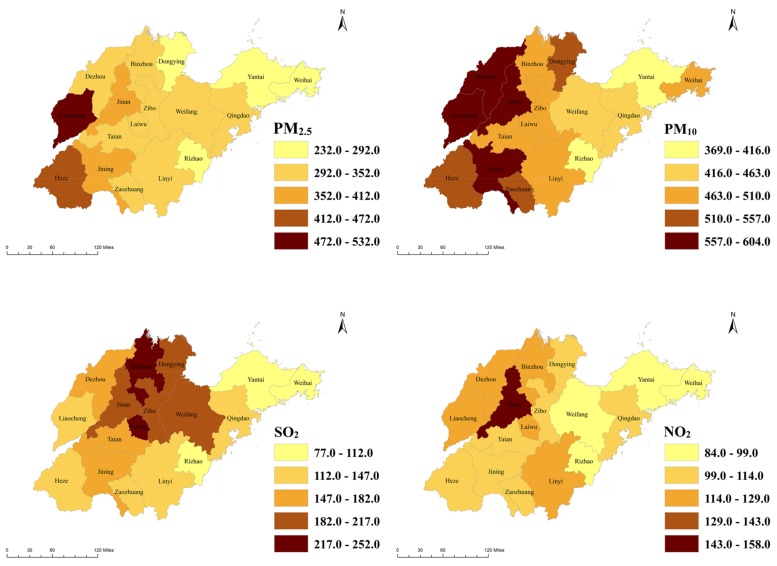
The daily maximum air pollution levels in the 17 cities of the Shandong Province.

Table A1, Table A2, Table A3, Table A4, Table A5, Table A6 and Table A7 in Appendix A present the minimum, maximum, 25% percentile, 50% percentile, 75% percentile, and mean values of the air pollution levels and meteorological factors from 1 January 2015 to 31 December 2016 in the 17 cities. Figure 2 shows the daily maximum air pollution levels in the 17 cities of the Shandong Province. Most of the daily maximum values of PM_2.5_, PM_10_, and NO_2_ in the 17 cities were 2–5 times that of China’s ambient air quality secondary standards (PM_2.5_: 75 μg/m^3^, PM_10_: 150 μg/m^3^, SO_2_: 150 μg/m^3^, and NO_2_: 80 μg/m^3^) [41], but the daily maximum concentrations of SO_2_ in 8 cities (Heze, Liaocheng, Linyi, Qingdao, Rizhao, Weihai, Ynatai, and Zaozhuang) were within the standard. There was an obvious spatial heterogeneity among the distribution of air pollution such that the air pollution concentrations in the eastern coastal cities like Qingdao, Yantai, Weihai, and Rizhao were generally lower than the measurements in the noncoastal cities.

**Figure 3:**
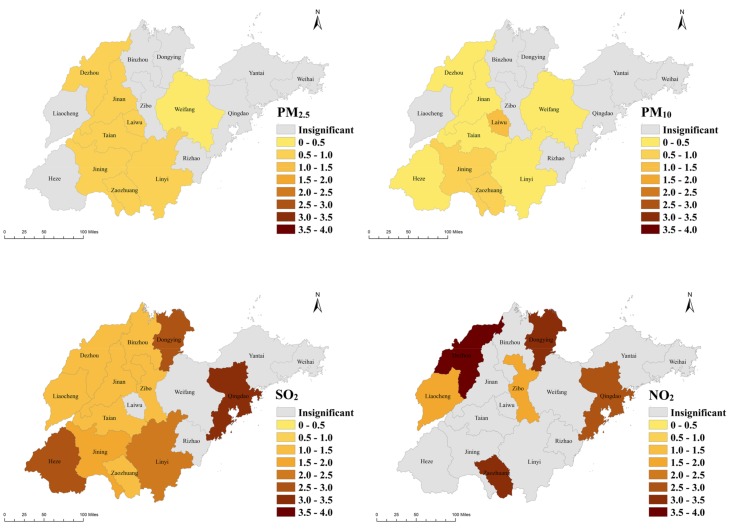
The percentage change in the relative risk of the total COPD hospitalizations per 10-μg/m^3^ increase in air pollution levels in the 17 cities of the Shandong province.

**Figure 4:**
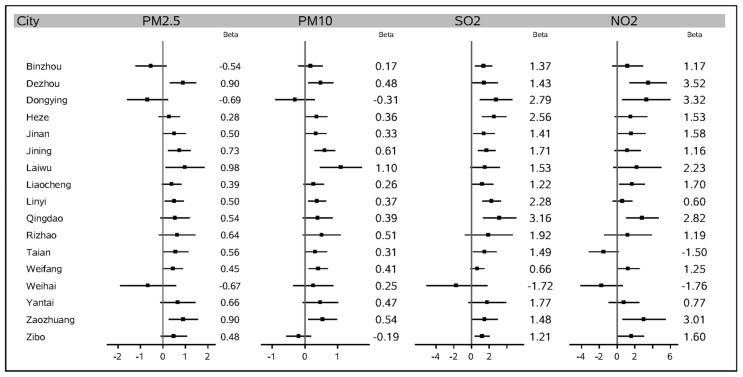
A forest plot of the percentage change in the relative risk of the total COPD hospitalizations with a 95% confidence interval (CI) per 10-μg/m^3^ increase in the air pollution levels in the 17 cities of the Shandong province.

Table 2 outlines the most statistically significant (*p* < 0.05) effect estimates and corresponding 95% confidence intervals (CIs) of the percentage changes of relative risk (RR) for the total COPD admissions per 10-μg/m^3^ increase in PM_2.5_, PM_10_, SO_2_, and NO_2_ concentrations for 0–6 day lags in Shandong. There were significant associations between PM_2.5_ and the total COPD admissions in 8 cities: Dezhou, Jinan, Jining, Laiwu, Linyi, Taian, Weifang, and Zaozhuang. The most severe effect was 0.978 (0.105, 1.858) by cause per 10-μg/m^3^ increase in PM_2.5_ in the city of Laiwu. The increase in PM_10_ concentrations led to a trend very similar to PM_2.5_, having a significant effect on the total admissions in 9 noncoastal cities, the largest one being 1.099 (0.458, 1.743), also in Laiwu. Furthermore, a 10-μg/m^3^ increase of SO_2_ was significantly associated with the increase of the total COPD hospital admissions in 12 cities, including two coastal cities: Dongying and Qingdao. Specifically, Qingdao had the most serious health effect: 3.164 (1.302, 5.059). Finally, the increase in NO_2_ levels had significant effects on hospitalizations in only 6 cities: Dezhou, Dongying, Liaocheng, Qingdao, Zaozhuang, and Zibo. The largest significant effect caused by NO_2_ in terms of the percentage change in the relative risk of hospitalizations for total patients was 3.515 (1.394, 5.590). This was in the city of Dezhou. These results for the total number of patients are also shown in the statistical map of Figure 3. The region colored in gray represents an insignificant effect in the area, while regions with a yellow to dark brown color demonstrate a significant percentage change in the relative risk of total COPD hospitalizations per 10-μg/m^3^ increase in that area. Figure 4 gives a forest plot of these results.

The health effects of air pollution were small in some cities with high pollution levels. From Table A1 and Table A2, we observed the highest levels of fine particulate matter (PM_2.5_ and PM_10_) occurred in the city of Liaocheng, which is located in the west of the province. However, the effects of PM_2.5_ and PM_10_ on the COPD hospital admissions in Liaocheng were 0.388 (−0.053, 0.830) and 0.262 (−0.052, 0.576), respectively; neither was significant. Similarly, during the study period, the most SO_2_- and NO_2_-polluted city was Zibo, located in the middle of the province. Although the effects of SO_2_ and NO_2_ were significant in Zibo (1.210 (0.375, 2.052) and 1.602 (0.130, 3.053), respectively), they were the weakest significant values among all the significant effects in the province. In contrast with this, SO_2_ and NO_2_ concentrations in Qingdao were relatively low, but the health effects from them were very strong: 3.164 (1.302, 5.059) and 2.824 (1.004, 4.677), respectively. In particular, the health effect caused by SO_2_ was the strongest among all the cities in Shandong.

Table A8, Table A9, Table A10 and Table A11 in Appendix A presents the results of the subgroup analysis. Regarding gender, stronger effects were observed for females. For example, the SO_2_ effect on male patients in Zaozhuang was insignificant: 1.279 (−0.346, 2.931), while the effect on female patients was significant: 2.570 (0.517, 4.666). Regarding age, although the effects on the aged patients were generally stronger, the effects on the younger patients were significant in some cities while the ones on older patients were not. For example, the PM_2.5_ effect on aged patients in Weifang was insignificant: 0.463 (−0.105, 1.035), but the effect on patients under 65 years of age was significant: 0.915 (0.226, 1.608).

## 4. Discussion

In this study, we examined the relationship between four air pollution (PM_2.5_, PM_10_, SO_2_, NO_2_) levels and the daily changes in COPD hospital admissions in 17 cities all over the Shandong province in China. We observed significant associations between ambient air pollution and COPD hospital admissions in Shandong. Epidemiological studies require reliable datasets. Therefore, for the health data, we adopted records from tertiary-level and secondary-level hospitals. All the patients went through a rigorous diagnosis and treatment during the admission time; hence, the accuracy of diagnosis can be guaranteed. Moreover, air pollution and meteorological data were collected from national monitoring stations. These datasets have the strength of analyzing the regional data using the national standard, avoiding the potential for publication bias in which positive findings are selectively reported.

From the results, we can see that the associations between air pollution and COPD hospitalizations were spatially heterogeneous across the province and that air pollution had obvious adverse health effects in the middle and west of the province. The cities in this area contained a large area of heavily polluting industrial sectors such as coal mining, the chemical industry, and the heavy industry. For example, Laiwu has the largest steel factory in the province, which made its PM_10_ and PM_2.5_ effects the strongest in the province. Geographical factors are also decisive. A large part of the middle and west of the Shandong province is hilly. The emitted ambient pollution is hard to blow away from these areas, consequently leading to a substantial exposure of the surrounding population to air pollution. Although the air pollution effects were much weaker in the coastal cities, SO_2_ and NO_2_ concentrations had significant influences on the hospitalizations in two coastal cities: Qingdao and Dongying. The most prominent source of NO_2_ and SO_2_ are fossil fuels burning from the industrial process with significant amounts of secondary aerosol, and the next most prominent sources are vehicle and domestic heating system exhaust emissions [42]. Qingdao is one of the most prosperous cities in China. It has the largest number of vehicles in Shandong: 2.3 million vehicles by the end of 2016, while there were only 1.8 million in the capital city of Jinan. Next, Dongying has the third largest oil field in China; the burning from the oil refinery process could be a major source of the SO_2_ and NO_2_ emissions which strongly affected the COPD hospital admissions.

We also found the effects of air pollution were small in some cities with high pollution levels. This could be caused by the active avoidance to exposure from the people living in heavily polluted cities, such as reducing outdoor activities or wearing masks. In addition, the body functions, especially the respiratory process, may have adjusted themselves to the long-term exposure to high-level pollution, hence, becoming less sensitive to toxic components. These results suggest that the studies focusing on a particular city may be unable to provide enough information for estimating the true effects, possibly even leading to biased significant results.

In the subgroup analysis, the relative risks of exposure to all of the pollutants for females were stronger for COPD. This is consistent with the results from previous studies that suggested that females were more vulnerable to ambient air pollution [33,43]. Furthermore, although the significant effects on the patients over 65 years of age were widespread in more cities, they were not overwhelmingly greater than the effects on younger patients. A possible explanation may be that the younger people would have more outdoor activities and are more likely to be exposed to air pollution if they were in the construction, manufacturing, or coal mining industries.

There are limitations in our analysis. Information on ambient concentrations in each city often comes from a number of monitoring sites, each of which may be subject to measurement errors and may contain periods of missing data. There is supposed to be a true underlying pollution surface which will form the basis of the exposures experienced by the population at risk. However, this surface is not directly observable and instead, measurements are taken at locations over space and time. The main limitation of this study is the lack of precise exposure estimates. Our exposure modeling approach is to simply take the average over all the air pollution data collected at the monitoring stations in the same area. This method may eliminate the spatial and temporal features of the ambient air pollution data; hence, the corresponding percentage change is not adequate. We need to apply a more sophisticated modeling method to the air pollution data in future studies.

## 5. Conclusions

This study assessed the associations between four types of air pollution and COPD hospital admissions using the daily time series data from 17 cities in the Shandong province, China. Our analysis showed that air pollution was significantly associated with adverse health outcomes. We found that the air pollution health effects on COPD were substantially impacted by the geographical factors and pollution type, such that air pollution had weaker impacts on COPD hospital admissions in coastal cities. Air pollution also had a stronger adverse effect on females. These results can help policymakers to determine the region-specific prevention measures for specific types of air pollution.

## Figures and Tables

**Table 1:** Descriptive statistics of the daily hospital admissions grouped by sex and age in the 17 cities of the Shandong province (from 1 January 2015 to 31 December 2016).

Caption	Total	Male	Female	≥65	<65
Binzhou	11,103	5700	5403	8972	2131
Dezhou	6288	3134	3154	4780	1508
Dongying	4026	2299	1727	3320	706
Heze	16,055	11,007	5048	12,593	3462
Jinan	13,711	8383	5328	10,707	3004
Jining	19,493	12,884	6609	14,611	4882
Laiwu	2823	1800	1023	2227	596
Liaocheng	15,368	9632	5736	11,783	3585
Linyi	25,508	13,830	11,678	18,402	7106
Qingdao	15,797	9613	6184	11,695	4102
Rizhao	5675	3118	2557	4128	1547
Taian	10,752	7052	3700	7709	3043
Weifang	26,653	15,832	10,821	19,439	7214
Weihai	7210	4612	2598	4929	2281
Yantai	11,848	7738	4110	8785	3063
Zaozhuang	12,623	7896	4727	9537	3086
Zibo	11,025	6848	4177	8822	2203
Total	215,958	131,378	84,580	162,439	53,519

**Table 2:** The percentage change in the relative risk of the total COPD hospitalizations and its 95% confidence interval (CI) per 10-μg/m^3^ increase in air pollutants in the 17 cities of the Shandong province, where * indicates statistically significant estimates (*p* < 0.05).

City	PM_2.5_	Lag	PM_10_	Lag	SO_2_	Lag	NO_2_	Lag
Binzhou	−0.541 (−1.23, 0.153)	0	0.172 (−0.202, 0.548)	3	1.374 (0.383, 2.356) *	5	1.168 (−0.555, 2.921)	3
Dezhou	0.895 (0.312, 1.474) *	2	0.481 (0.095, 0.865) *	2	1.427 (0.089, 2.970) *	0	3.515 (1.394, 5.590) *	2
Dongying	−0.69 (−1.604, 0.232)	3	−0.305 (−0.902, 0.294)	3	2.787 (0.926, 4.612) *	0	3.316 (0.573, 5.984) *	0
Heze	0.279 (−0.203, 0.764)	1	0.357 (0.022, 0.694) *	3	2.558 (1.186, 3.948) *	2	1.534 (−0.301, 3.402)	3
Jinan	0.501 (0.003, 1.018) *	4	0.332 (0.008, 0.658) *	4	1.405 (0.203, 2.621) *	3	1.583 (−0.025, 3.218)	0
Jining	0.729 (0.232, 1.229) *	2	0.605 (0.285, 0.925) *	2	1.713 (0.734, 2.701) *	4	1.164 (−0.309, 2.659)	4
Laiwu	0.978 (0.105, 1.858) *	3	1.099 (0.458, 1.743) *	3	1.530 (−0.132, 3.219)	2	2.233 (−0.427, 4.965)	2
Liaocheng	0.388 (−0.053, 0.830)	0	0.262 (−0.052, 0.576)	0	1.217 (0.036, 2.485) *	3	1.697 (0.247, 3.126) *	5
Linyi	0.501 (0.081, 0.922) *	1	0.372 (0.099, 0.646) *	1	2.276 (1.234, 3.328) *	2	0.599 (−0.511, 1.721)	2
Qingdao	0.536 (−0.111, 1.188)	0	0.388 (−0.064, 0.843)	0	3.164 (1.302, 5.059) *	0	2.824 (1.004, 4.677) *	0
Rizhao	0.635 (−0.179, 1.455)	2	0.510 (−0.065, 1.088)	3	1.916 (−0.772, 4.677)	4	1.187 (−1.447, 3.890)	0
Taian	0.561 (0.004, 1.140) *	2	0.314 (0.018, 0.678) *	3	1.490 (0.167, 2.830) *	2	−1.501 (−3.191, 0.218)	5
Weifang	0.448 (0.007, 0.890) *	2	0.406 (0.109, 0.704) *	5	0.656 (−0.156, 1.475)	2	1.245 (−0.052, 2.559)	2
Weihai	−0.667 (−1.907, 0.590)	0	0.254 (−0.361, 0.873)	2	−1.717 (−5.102, 1.789)	0	−1.756 (−4.111, 0.657)	2
Yantai	0.658 (−0.120, 1.442)	5	0.470 (−0.069, 1.012)	2	1.768 (−0.357, 3.938)	2	0.765 (−0.948, 2.508)	2
Zaozhuang	0.904 (0.256, 1.555) *	1	0.542 (0.103, 0.983) *	1	1.484 (0.015, 2.973) *	5	3.013 (0.596, 5.488) *	6
Zibo	0.480 (−0.102, 1.066)	3	−0.189 (−0.567, 0.190)	4	1.210 (0.375, 2.052) *	3	1.602 (0.130, 3.053) *	6

## Supplementary Materials

Supplementary File 1

## Appendix A

**Table A1:** Descriptive statistics of the daily mean PM_2.5_ levels (μg/m^3^) in the 17 cities of the Shandong province (from 1 January 2015 to 31 December 2016).

Caption	Min	25%	50%	75%	Max	Mean
Binzhou	7.7	42.2	65.4	96.0	336.7	75.1
Dezhou	5.8	42.0	67.6	98.6	307.1	79.4
Dongying	7.0	31.0	52.0	79.3	269.1	60.4
Heze	9.2	48.7	70.1	108.2	427.4	85.8
Jinan	13.3	45.0	63.5	95.3	382.1	76.0
Jining	11.8	42.9	60.8	93.1	366.1	74.1
Laiwu	16.1	45.7	68.9	101.2	323.6	80.8
Liaocheng	9.7	50.9	74.8	112.3	529.8	89.7
Linyi	11.2	34.4	56.2	89.3	345.0	71.5
Qingdao	7.7	23.6	37.2	57.9	308.4	46.4
Rizhao	4.8	24.0	40.4	66.0	279.3	52.3
Taian	12.1	37.6	55.1	81.9	346.2	67.0
Weifang	9.7	38.2	56.5	84.5	336.0	67.4
Weihai	2.3	19.0	27.9	46.2	232.3	35.3
Yantai	5.1	20.4	31.5	50.8	245.1	39.5
Zaozhuang	8.6	44.1	67.4	100.4	300.6	80.0
Zibo	16.0	42.9	61.1	86.8	335.8	70.7

**Table A2:** Descriptive statistics of the daily mean PM_10_ levels (μg/m^3^) in the 17 cities of the Shandong province (from 1 January 2015 to 31 December 2016).

Caption	Min	25%	50%	75%	Max	Mean
Binzhou	13.4	70.6	107.7	157.1	486.9	121.1
Dezhou	9.0	81.7	122.4	177.2	584.3	138.0
Dongying	15.9	61.6	94.0	142.8	546.6	108.6
Heze	13.7	88.7	125.8	184.6	549.5	143.6
Jinan	21.1	91.3	125.0	176.1	600.3	141.9
Jining	9.0	75.8	105.9	155.1	589.2	123.4
Laiwu	12.1	83.3	120.0	159.0	471.6	129.6
Liaocheng	10.4	97.2	132.9	186.4	577.9	151.0
Linyi	19.2	73.2	117.4	170.9	499.5	134.2
Qingdao	19.6	51.8	72.9	107.0	454.7	86.2
Rizhao	8.5	45.6	73.8	117.9	414.7	89.5
Taian	14.1	71.9	105.1	146.1	486.1	117.8
Weifang	27.8	84.2	113.9	162.3	460.8	128.0
Weihai	8.7	37.8	51.6	78.5	464.5	64.3
Yantai	13.6	40.2	57.1	83.9	369.4	67.0
Zaozhuang	23.1	90.3	129.9	178.8	542.6	142.4
Zibo	18.7	80.4	117.5	154.3	489.4	125.6

**Table A3:** Descriptive statistics of the daily mean SO_2_ levels (μg/m^3^) in the 17 cities of the Shandong province (from 1 January 2015 to 31 December 2016).

Caption	Min	25%	50%	75%	Max	Mean
Binzhou	6.2	24.3	38.7	60.3	248.5	46.2
Dezhou	3.4	18.7	28.1	42.7	181.2	35.0
Dongying	3.0	24.1	37.3	56.1	196.0	43.4
Heze	9.7	23.1	32.8	46.7	135.4	36.9
Jinan	8.2	20.5	31.1	49.7	185.2	39.3
Jining	9.2	29.0	42.5	63.9	166.4	49.1
Laiwu	2.4	27.8	43.1	64.2	217.5	51.1
Liaocheng	3.7	20.0	31.1	47.0	123.4	35.9
Linyi	3.2	14.2	26.5	42.5	139.0	32.3
Qingdao	5.0	15.2	20.1	27.6	130.3	24.0
Rizhao	4.3	10.7	16.0	24.6	110.0	20.2
Taian	7.4	21.5	32.1	47.9	149.9	37.7
Weifang	6.7	18.8	29.1	50.0	213.3	38.9
Weihai	1.2	9.1	12.8	17.8	92.5	15.4
Yantai	3.6	11.4	16.8	25.5	77.3	20.1
Zaozhuang	10.4	25.8	40.8	57.6	136.0	44.1
Zibo	7.0	36.9	55.5	74.6	208.2	60.8

**Table A4:** Descriptive statistics of the daily mean NO_2_ levels (μg/m^3^) in the 17 cities of the Shandong province (from 1 January 2015 to 31 December 2016).

Caption	Min	25%	50%	75%	Max	Mean
Binzhou	7.8	24.3	35.2	49.1	124.4	38.9
Dezhou	5.7	23.6	32.2	43.7	117.7	35.9
Dongying	2.7	19.4	30.5	44.9	103.1	34.0
Heze	5.8	25.5	34.3	46.5	102.4	37.6
Jinan	10.9	31.3	42.5	57.7	155.1	45.9
Jining	8.0	29.2	40.6	53.6	106.2	42.9
Laiwu	8.2	31.9	44.4	58.6	116.1	47.0
Liaocheng	4.6	27.5	38.0	50.2	115.8	41.4
Linyi	8.5	29.6	41.9	57.2	125.7	45.1
Qingdao	7.1	21.6	30.8	41.1	107.0	32.7
Rizhao	3.9	19.5	30.6	44.1	98.2	33.1
Taian	7.5	28.4	38.9	49.4	107.5	40.3
Weifang	8.7	22.6	31.9	44.1	96.5	35.0
Weihai	3.8	13.5	19.7	27.9	92.7	22.1
Yantai	8.0	20.7	29.1	39.8	84.6	31.8
Zaozhuang	8.9	21.7	29.2	38.8	101.4	31.4
Zibo	13.7	38.7	48.8	58.3	102.4	49.7

**Table A5:** Descriptive statistics of the daily mean temperature (°C) in the 17 cities of the Shandong province (from 1 January 2015 to 31 December 2016).

Caption	Min	25%	50%	75%	Max	Mean
Binzhou	−15.3	3.8	16.1	23.3	31.7	13.9
Dezhou	−14.3	3.6	15.4	22.9	31.8	13.5
Dongying	−13.2	3.9	16.5	24.0	32.6	14.4
Heze	−9.2	5.8	16.4	23.3	31.3	14.7
Jinan	−12.4	6.0	17.4	24.2	33.0	15.3
Jining	−10.7	4.8	16.2	23.4	32.5	14.5
Laiwu	−13.1	4.0	15.7	22.6	32.0	13.7
Liaocheng	−10.9	5.2	16.4	23.5	31.5	14.5
Linyi	−11.7	5.5	16.6	23.7	32.5	14.8
Qingdao	−11.5	5.5	15.0	21.9	29.4	13.8
Rizhao	−11.4	5.7	14.9	22.2	31.4	14.1
Taian	−22.8	−1.6	8.4	14.8	22.7	6.7
Weifang	−13.0	4.5	16.3	23.6	33.2	14.3
Weihai	−11.8	4.9	14.9	22.5	30.6	13.8
Yantai	−11.9	3.7	15.0	22.6	31.4	13.3
Zaozhuang	−11.0	5.8	16.4	23.4	32.2	14.7
Zibo	−13.1	5.2	17.3	23.9	32.9	14.9

**Table A6:** Descriptive statistics of the daily mean relative humidity (%) in the 17 cities of the Shandong province (from 1 January 2015 to 31 December 2016).

Caption	Min	25%	50%	75%	Max	Mean
Binzhou	25	51	64	77	98	63.7
Dezhou	26	57	70	82	100	69.3
Dongying	23	49	62	73	98	61.7
Heze	30	60	71	83	98	70.2
Jinan	19	42	55	72	100	57.4
Jining	32	61	73	82	100	70.9
Laiwu	23	49	63	77	97	62.3
Liaocheng	24	54	67	79	98	66.2
Linyi	24	53	68	78	99	65.5
Qingdao	21	57	72	83	100	69.6
Rizhao	22	55	72	85	99	69.1
Taian	7	45	67	87.5	100	65.9
Weifang	24	51	64	74	96	62.6
Weihai	18	52	64	76	97	63.1
Yantai	22	54	66	76	96	64.2
Zaozhuang	28	63	75	83	98	72.9
Zibo	20	44	57	72	98	58

**Table A7:** Descriptive statistics of the daily mean wind speed (m/s) in the 17 cities of the Shandong province (from 1 January 2015 to 31 December 2016).

Caption	Min	25%	50%	75%	Max	Mean
Binzhou	0.4	1.4	1.9	2.6	6.4	2.1
Dezhou	0.3	1.5	2.2	3.1	7.2	2.4
Dongying	0.5	1.6	2.0	2.6	5.4	2.2
Heze	0.4	1.5	2.1	2.9	6.3	2.3
Jinan	0.2	1.5	2.0	2.8	6.4	2.3
Jining	0.2	1.0	1.4	1.9	4.2	1.5
Laiwu	0.0	1.2	1.6	2.1	5.7	1.7
Liaocheng	0.1	1.3	1.8	2.6	7.5	2.0
Linyi	0.2	1.1	1.5	1.9	5.3	1.6
Qingdao	1.0	2.3	2.9	3.7	8.4	3.2
Rizhao	0.8	2.2	2.9	3.7	11.3	3.1
Taian	1.0	4.1	5.9	7.7	20.5	6.2
Weifang	0.2	1.4	1.9	2.5	5.9	2.0
Weihai	0.5	2.1	3.0	4.4	9.6	3.4
Yantai	0.7	2.0	2.7	3.8	8.7	3.0
Zaozhuang	0.3	1.3	1.7	2.3	6.0	1.8
Zibo	0.3	1.4	1.8	2.7	7.3	2.2

**Table A8:** Percentage changes of the relative risk of male COPD hospitalizations and their 95% confidence interval (CI) per 10-μg/m^3^ increase in air pollutants in the 17 cities of the Shandong province, where * indicates statistically significant estimates (*p* < 0.05).

Caption	PM_2.5_	PM_10_	SO_2_	NO_2_
Binzhou	−0.512 (−1.24, 0.221)	−0.301 (−0.892, 0.294)	−1.149 (−2.420, 0.139)	−1.540 (−3.690, 0.658)
Dezhou	0.807 (0.051, 1.557) *	0.612 (0.105, 1.117) *	1.824 (−0.278, 3.969)	3.467 (0.689, 6.167) *
Dongying	−0.681 (−1.834, 0.485)	−0.369 (−1.130, 0.398)	2.142 (0.204, 4.118) *	−1.422 (−4.223, 1.460)
Heze	0.199 (−0.355, 0.755)	0.315 (−0.067, 0.700)	2.407 (0.834, 4.004) *	−1.230 (−3.470, 1.062)
Jinan	0.533 (−0.097, 1.167)	0.373 (−0.017, 0.764)	1.167 (−0.279, 2.633)	1.939 (0.052, 3.924) *
Jining	0.754 (0.182, 1.330) *	0.624 (0.257, 0.993) *	1.796 (0.666, 2.938) *	1.652 (−0.781, 4.145)
Laiwu	0.989 (−0.099, 2.090)	0.650 (−0.143, 1.450)	1.989 (−0.01, 4.029)	3.134 (−0.101, 6.474)
Liaocheng	0.492 (−0.030, 1.016)	−0.247 (−0.619, 0.127)	1.235 (−0.256, 2.747)	−1.928 (−3.946, 0.133)
Linyi	0.628 (0.141, 1.118) *	0.404 (0.088, 0.722) *	2.033 (0.817, 3.263) *	0.752 (−0.535, 2.056)
Qingdao	0.425 (−0.291, 1.146)	0.391 (−0.076, 0.859)	2.933 (0.609, 5.311) *	2.715 (0.481, 4.998) *
Rizhao	0.631 (−0.608, 1.886)	0.574 (−0.329, 1.485)	2.840 (−0.614, 6.413)	2.175 (−1.222, 5.689)
Taian	0.716 (−0.035, 1.473)	0.271 (−0.165, 0.709)	1.448 (−0.171, 3.094)	0.851 (−1.346, 3.096)
Weifang	0.414 (−0.097, 0.929)	0.360 (0.016, 0.706) *	0.612 (−0.299, 1.532)	0.744 (−0.755, 2.266)
Weihai	0.282 (−1.049, 1.631)	−0.165 (−0.914, 0.590)	−1.844 (−5.290, 1.727)	−2.375 (−5.177, 0.510)
Yantai	0.732 (−0.185, 1.657)	0.538 (−0.092, 1.173)	2.216 (−0.314, 4.809)	1.011 (−0.993, 3.056)
Zaozhuang	0.747 (0.041, 1.457) *	0.501 (0.026, 0.979) *	1.279 (−0.346, 2.931)	2.034 (−0.571, 4.707)
Zibo	0.627 (−0.084, 1.343)	0.329 (−0.200, 0.862)	1.050 (0.027, 2.082) *	1.096 (−1.001, 3.238)

**Table A9:** Percentage changes of the relative risk of female COPD hospitalizations and their 95% confidence interval (CI) per 10-μg/m^3^ increase in air pollutants in the 17 cities of the Shandong province, where * indicates statistically significant estimates (*p* < 0.05).

Caption	PM_2.5_	PM_10_	SO_2_	NO_2_
Binzhou	−0.796 (−1.723, 0.140)	0.390 (−0.112, 0.895)	1.616 (0.273, 2.978) *	2.889 (0.542, 5.291) *
Dezhou	1.033 (0.265, 1.795) *	0.641 (0.139, 1.140) *	2.196 (0.211, 4.220) *	3.981 (1.204, 6.680) *
Dongying	−1.328 (−2.664, 0.027)	−0.321 (−1.186, 0.551)	3.681 (1.000, 6.290) *	4.568 (1.312, 7.716) *
Heze	0.897 (0.120, 1.679) *	0.753 (0.206, 1.303) *	2.667 (0.609, 4.767) *	3.086 (0.461, 5.779) *
Jinan	1.132 (0.452, 1.818) *	0.528 (0.087, 0.971) *	1.840 (0.264, 3.440) *	1.969 (0.099, 3.745) *
Jining	0.773 (0.067, 1.485) *	0.617 (0.161, 1.075) *	2.025 (0.619, 3.450) *	1.804 (0.105, 3.531) *
Laiwu	1.826 (0.442, 3.229) *	1.916 (0.893, 2.950) *	2.819 (0.147, 5.564) *	4.186 (−0.074, 8.628)
Liaocheng	−0.325 (−0.845, 0.198)	0.373 (0.004, 0.743) *	1.228 (−0.551, 3.040)	2.082 (0.361, 3.772) *
Linyi	0.672 (0.202, 1.144) *	0.426 (0.119, 0.734) *	2.551 (1.291, 3.827) *	1.669 (0.037, 3.326) *
Qingdao	0.767 (−0.147, 1.689)	0.561 (−0.079, 1.205)	3.501 (0.930, 6.137) *	2.798 (0.207, 5.456) *
Rizhao	0.774 (−0.285, 1.844)	0.535 (−0.234, 1.310)	−3.286 (−6.891, 0.458)	−1.864 (−4.632, 0.984)
Taian	1.030 (0.197, 1.871) *	0.575 (0.007, 1.147) *	2.444 (0.500, 4.426) *	2.703 (0.215, 5.129) *
Weifang	0.713 (0.101, 1.329) *	0.561 (0.147, 0.977) *	1.071 (0.018, 2.135) *	1.944 (0.231, 3.687) *
Weihai	−1.775 (−3.721, 0.211)	0.625 (−0.353, 1.613)	4.056 (−0.496, 8.816)	−2.327 (−6.040, 1.534)
Yantai	1.719 (0.549, 2.903) *	1.121 (0.283, 1.967) *	4.304 (0.952, 7.767) *	−1.751 (−4.356, 0.925)
Zaozhuang	1.071 (0.213, 1.937) *	0.712 (0.134, 1.292) *	2.570 (0.517, 4.666) *	4.861 (1.480, 8.354) *
Zibo	−0.782 (−1.624, 0.066)	−0.450 (−0.993, 0.096)	1.398 (0.203, 2.607) *	2.180 (−0.320, 4.742)

**Table A10:** Percentage changes of the relative risk of COPD hospitalizations for patients aged over 65 years and their 95% confidence interval (CI) per 10-μg/m^3^ increase in air pollutants in the 17 cities of the Shandong province, where * indicates statistically significant estimates (*p* < 0.05).

Caption	PM_2.5_	PM_10_	SO_2_	NO_2_
Binzhou	−0.691 (−1.447, 0.071)	−0.409 (−0.914, 0.099)	1.185 (0.100, 2.259) *	1.256 (−0.624, 3.173)
Dezhou	1.065 (0.425, 1.701) *	0.569 (0.144, 0.992) *	1.659 (0.019, 3.272) *	4.125 (1.805, 6.390) *
Dongying	−0.714 (−1.695, 0.278)	0.400 (−0.276, 1.08)	2.548 (0.545, 4.510) *	2.723 (0.333, 5.056) *
Heze	0.606 (0.119, 1.09) *	0.419 (0.064, 0.776) *	2.891 (1.439, 4.363) *	2.389 (0.448, 4.367) *
Jinan	0.728 (0.157, 1.295) *	0.348 (0.001, 0.697) *	1.347 (0.064, 2.646) *	1.843 (0.120, 3.596) *
Jining	0.620 (0.062, 1.182) *	0.646 (0.303, 0.991) *	1.711 (0.654, 2.779) *	1.723 (−0.134, 3.614)
Laiwu	0.927 (−0.082, 1.946)	1.135 (0.394, 1.881) *	2.030 (0.124, 3.972) *	2.619 (−0.460, 5.793)
Liaocheng	0.416 (−0.053, 0.887)	−0.239 (−0.522, 0.046)	1.250 (−0.103, 2.621)	2.063 (0.502, 3.598) *
Linyi	0.476 (0.017, 0.938) *	0.351 (0.051, 0.652) *	2.365 (1.222, 3.520) *	0.930 (−0.536, 2.417)
Qingdao	0.655 (−0.078, 1.393)	0.504 (−0.008, 1.018)	3.186 (1.065, 5.352) *	3.218 (1.150, 5.330) *
Rizhao	−0.532 (−1.490, 0.435)	0.449 (−0.196, 1.098)	2.163 (−0.845, 5.262)	−1.107 (−3.428, 1.270)
Taian	0.785 (0.148, 1.426) *	0.431 (0.002, 0.865) *	1.805 (0.325, 3.307) *	1.498 (−0.448, 3.482)
Weifang	0.463 (−0.105, 1.035)	0.388 (0.043, 0.734) *	0.559 (−0.325, 1.451)	−1.100 (−2.478, 0.299)
Weihai	−0.565 (−1.997, 0.887)	0.218 (−0.493, 0.933)	−2.280 (−6.180, 1.782)	−1.765 (−4.492, 1.040)
Yantai	0.573 (−0.304, 1.456)	0.669 (0.050, 1.291) *	1.995 (−0.389, 4.436)	−1.428 (−3.290, 0.470)
Zaozhuang	0.847 (0.148, 1.55) *	0.484 (0.009, 0.961) *	1.766 (0.181, 3.377) *	3.761 (1.148, 6.442) *
Zibo	0.583 (−0.097, 1.267)	0.346 (−0.092, 0.787)	1.044 (0.126, 1.971) *	1.137 (−0.543, 2.845)

**Table A11:** Percentage changes of the relative risk of COPD hospitalizations for patients aged under 65 years and their 95% confidence interval (CI) per 10-μg/m^3^ increase in air pollutants in the 17 cities of the Shandong province, where * indicates statistically significant estimates (*p* < 0.05).

Caption	PM_2.5_	PM_10_	SO_2_	NO_2_
Binzhou	−0.988 (−2.092, 0.128)	−0.635 (−1.369, 0.104)	2.391 (0.395, 4.347) *	3.063 (−0.921, 7.207)
Dezhou	1.395 (0.320, 2.458) *	1.103 (0.390, 1.810) *	2.134 (−0.595, 4.938)	5.770 (2.022, 9.374) *
Dongying	−1.874 (−4.052, 0.352)	−0.785 (−2.115, 0.562)	−2.630 (−6.456, 1.352)	−4.267 (−9.071, 0.791)
Heze	0.465 (−0.496, 1.434)	0.425 (−0.178, 1.032)	1.804 (−0.725, 4.397)	−3.105 (−6.244, 0.140)
Jinan	0.671 (−0.216, 1.566)	0.460 (−0.102, 1.025)	1.400 (−0.683, 3.527)	−1.546 (−3.882, 0.847)
Jining	1.227 (0.419, 2.041) *	0.641 (0.117, 1.167) *	2.033 (0.429, 3.661) *	−1.926 (−4.304, 0.512)
Laiwu	1.492 (−0.193, 3.205)	1.011 (−0.222, 2.259)	3.206 (0.461, 6.027) *	7.241 (1.718, 13.064) *
Liaocheng	0.602 (−0.028, 1.236)	0.400 (−0.105, 0.908)	1.790 (−0.442, 4.071)	−0.933 (−3.473, 1.674)
Linyi	0.934 (0.340, 1.532) *	0.476 (0.061, 0.893) *	2.054 (0.468, 3.666) *	1.806 (0.140, 3.501) *
Qingdao	−0.924 (−1.994, 0.157)	−0.316 (−1.030, 0.403)	2.965 (−0.340, 6.380)	2.849 (0.144, 5.480) *
Rizhao	0.854 (−0.850, 2.587)	0.754 (−0.216, 1.733)	2.222 (−2.557, 7.235)	2.463 (−1.233, 6.297)
Taian	0.852 (−0.181, 1.896)	−0.413 (−1.034, 0.212)	1.710 (−0.516, 3.985)	−1.951 (−4.626, 0.800)
Weifang	0.915 (0.226, 1.608) *	0.711 (0.244, 1.181) *	−1.502 (−3.008, 0.027)	2.676 (0.610, 4.785) *
Weihai	−0.718 (−2.688, 1.291)	0.703 (−0.297, 1.713)	1.144 (−3.569, 6.087)	−2.033 (−5.888, 1.980)
Yantai	2.192 (0.843, 3.558) *	1.584 (0.656, 2.521) *	5.282 (1.384, 9.330) *	2.677 (−0.473, 5.926)
Zaozhuang	1.073 (0.006, 2.151) *	0.750 (0.034, 1.471) *	1.681 (−0.791, 4.213)	2.202 (−1.837, 6.406)
Zibo	−0.994 (−2.180, 0.207)	−0.780 (−1.591, 0.037)	1.606 (−0.108, 3.348)	−2.753 (−5.729, 0.318)

## References

[1] Katsouyanni K. (2003). Ambient air pollution and health. Br. Med. Bull..

[2] Brauer M., Freedman G., Frostad J., Van Donkelaar A., Martin R.V., Dentener F., Dingenen R.V., Estep K., Amini H., Apte J.S. (2015). Ambient air pollution exposure estimation for the global burden of disease 2013. Environ. Sci. Technol..

[3] Lelieveld J., Evans J.S., Fnais M., Giannadaki D., Pozzer A. (2015). The contribution of outdoor air pollution sources to premature mortality on a global scale. Nature.

[4] Raaschou-Nielsen O., Andersen Z.J., Beelen R., Samoli E., Stafoggia M., Weinmayr G., Hoffmann B., Fischer P., Nieuwenhuijsen M.J., Brunekreef B. (2013). Air pollution and lung cancer incidence in 17 European cohorts: Prospective analyses from the European Study of Cohorts for Air Pollution Effects (ESCAPE). Lancet Oncol..

[5] Brunekreef B., Holgate S.T. (2002). Air pollution and health. Lancet.

[6] World Health Organization (2011). Burden of Disease Attributable to Outdoor Air Pollution.

[7] Lim S.S., Vos T., Flaxman A.D., Danaei G., Shibuya K., Adair-Rohani H., AlMazroa M.A., Amann M., Anderson H.R., Andrews K.G. (2013). A comparative risk assessment of burden of disease and injury attributable to 67 risk factors and risk factor clusters in 21 regions, 1990–2010: A systematic analysis for the Global Burden of Disease Study 2010. Lancet.

[8] Stieb D.M., Judek S., Burnett R.T. (2002). Meta-Analysis of Time-Series Studies of Air Pollution and Mortality: Effects of Gases and Particles and the Influence of Cause of Death, Age, and Season. J. Air Waste Manag. Assoc..

[9] Sun Q., Hong X., Wold L.E. (2010). Cardiovascular Effects of Ambient Particulate Air Pollution Exposure. Circulation.

[10] Dominici F., Peng R.D., Bell M.L., Pham L., McDermott A., Zeger S.L., Samet J.M. (2006). Fine particulate air pollution and hospital admission for cardiovascular and respiratory diseases. JAMA.

[11] Brook R.D., Rajagopalan S., Pope C.A., Brook J.R., Bhatnagar A., Diez-Roux A.V., Holguin F., Hong Y., Luepker R.V., Mittleman M.A. (2010). Particulate matter air pollution and cardiovascular disease. Circulation.

[12] Schikowski T., Sugiri D., Ranft U., Gehring U., Heinrich J., Wichmann H.E., Krämer U. (2005). Long-term air pollution exposure and living close to busy roads are associated with COPD in women. Respir. Res..

[13] Rabe K.F., Hurd S., Anzueto A., Jones P.W., Vogelmeier C., Anzueto A., Barnes P.J., Fabbri L.M., Martinez F.J., Nishimura M. (2013). Global strategy for the diagnosis, management, and prevention of chronic obstructive pulmonary disease: GOLD executive summary. Am. J. Respir. Crit. Care Med..

[14] World Health Organization Chronic Obstructive Pulmonary Disease (COPD). http://www.who.int/respiratory/copd/en/.

[15] World Health Organization (2008). The Global Burden of Disease. 2004 Update.

[16] Chan C.K., Yao X. (2008). Air pollution in mega cities in China. Atmos. Environ..

[17] Rohde R.A., Muller R.A. (2015). Air pollution in China: Mapping of concentrations and sources. PLoS ONE.

[18] Huang R.J., Zhang Y., Bozzetti C., Ho K.F., Cao J.J., Han Y., Daellenbach K.R., Slowik J.G., Platt S.M., Canonaco F. (2014). High secondary aerosol contribution to particulate pollution during haze events in China. Nature.

[19] Cao J., Yang C., Li J., Chen R., Chen B., Gu D., Kan H. (2011). Association between long-term exposure to outdoor air pollution and mortality in China: A cohort study. J. Hazard. Mater..

[20] Chen R., Kan H., Chen B., Huang W., Bai Z., Song G., Pan G., CAPES Collaborative Group (2012). Association of Particulate Air Pollution with Daily Mortality: The China Air Pollution and Health Effects Study. Am. J. Epidemiol..

[21] Chen Z., Wang J.N., Ma G.X., Zhang Y.S. (2013). China tackles the health effects of air pollution. Lancet.

[22] Zhou M., He G., Liu Y., Yin P., Li Y., Kan H., Fan M., Xue A., Fan M. (2015). The associations between ambient air pollution and adult respiratory mortality in 32 major Chinese cities, 2006–2010. Environ. Res..

[23] Schwartz J., Morris R. (1995). Air pollution and hospital admissions for cardiovascular disease in Detroit, Michigan. Am. J. Epidemiol..

[24] Schwartz J. (1999). Air pollution and hospital admissions for heart disease in eight US counties. Epidemiology.

[25] Bell M.L., Ebisu K., Peng R.D., Samet J.M., Dominici F. (2009). Hospital admissions and chemical composition of fine particle air pollution. Am. J. Respir. Crit. Care Med..

[26] Peng R., Chang H., Bell M., McDermott A., Zeger S., Samet J., Dominici F. (2008). Coarse particulate matter air pollution and hospital admissions for cardiovascular and respiratory diseases among Medicare patients. JAMA.

[27] Samoli E., Atkinson R.W., Analitis A., Fuller G.W., Green D.C., Mudway I., Anderson H.R., Kelly F.J. (2016). Associations of short-term exposure to traffic-related air pollution with cardiovascular and respiratory hospital admissions in London, UK. Occup. Environ. Med..

[28] Atkinson R.W., Kang S., Anderson H.R., Mills I.C., Walton H.A. (2014). Epidemiological time series studies of PM_2.5_ and daily mortality and hospital admissions: A systematic review and meta-analysis. Thorax.

[29] Dockery D.W., Rich D.Q., Goodman P.G., Clancy L., Ohman-Strickland P., George P., Kotlov T. (2013). Effect of air pollution control on mortality and hospital admissions in Ireland. Res. Rep. Health Eff. Inst..

[30] Lanki T., Pekkanen J., Aalto P., Elosua R., Berqlind N., D’lppoliti D., Kulmala M., Nyberg F., Peters A., Picciotto S. (2006). Associations of traffic related air pollution levels with hospitalization for first acute myocardial infarction: The HEAPSS study. Occup. Environ. Med..

[31] Gan W.Q., FitzGerald J.M., Carlsten C., Sadatsafavi M., Brauer M. (2013). Associations of ambient air pollution with chronic obstructive pulmonary disease hospitalization and mortality. Am. J. Respir. Crit. Care Med..

[32] Xiong Q., Zhao W., Gong Z., Zhao W., Tang T. (2015). Fine Particulate Matter Pollution and Hospital Admissions for Respiratory Diseases in Beijing, China. Int. J. Environ. Res. Public Health.

[33] Tao Y., Mi S., Zhou S., Wang S., Xie X. (2014). Air pollution and hospital admissions for respiratory diseases in Lanzhou, China. Environ. Pollut..

[34] Zhang Z., Wang J., Chen L., Chen X., Sun G., Zhong N., Kan H., Lu W. (2014). Impact of haze and air pollution-related hazards on hospital admissions in Guangzhou, China. Environ. Sci. Pollut. Res..

[35] Chen R., Chu C., Tan J., Cao J., Song W., Xu X., Jiang C., Ma W., Yang C., Chen B. (2010). Ambient air pollution and hospital admission in Shanghai, China. J. Hazard. Mater..

[36] Xiang H., Mertz K.J., Arena V.C., Brink L.L., Xu X., Bi Y., Talbott E.O. (2013). Estimation of short-term effects of air pollution on stroke hospital admissions in Wuhan, China. PLoS ONE.

[37] Hastie T.J., Tibshirani R.J. (1990). Generalized additive models. Statistical Models in S.

[38] Wood S. (2006). Generalized Additive Models: An Introduction with R.

[39] Wahba G. (1990). Spline Models for Observational Data.

[40] Gu C. (2013). Smoothing Spline ANOVA Models.

[41] (2016). Ministry of Environmental Protection of the People’s Republic of China. http://kjs.mep.gov.cn/hjbhbz/bzwb/dqhjbh/dqhjzlbz/201203/t20120302_224165.shtml.

[42] World Health Organization (2006). Air Quality Guidelines: Global Update 2005: Particulate Matter, Ozone, Nitrogen Dioxide, and Sulfur Dioxide.

[43] Kan H., London S.J., Chen G., Zhang Y., Song G., Zhao N., Jiang L., Chen B. (2008). Season, sex, age, and education as modifiers of the effects of outdoor air pollution on daily mortality in shanghai, china: The public health and air pollution in Asia (papa) study. Environ. Health Perspect..

