# Supplementary material for: A Multicity Analysis of the Short-Term Effects of Air Pollution on the Chronic Obstructive Pulmonary Disease Hospital Admissions in Shandong, China
Source: Int J Environ Res Public Health. 2018 Apr 17;15(4):774. doi: 10.3390/ijerph15040774 (PMC5923816; doi:10.3390/ijerph15040774)
Supplement: Supplementary File 1 [file ijerph-15-00774-s001.pdf]

City	$PM_{2.5}$	Lag	PM_{10}	Lag	SO_2	Lag	NO_2	Lag
Binzhou	–0.541 (–1.23, 0.153)	0	0.172 (–0.202, 0.548)	3	1.374 (0.383, 2.356)*	5	1.168 (–0.555, 2.921)	3
Dezhou	0.895 (0.312, 1.474)*	2	0.481 (0.095, 0.865)*	2	1.427 (0.089, 2.970)*	0	3.515 (1.394, 5.590)*	2
Dongying	–0.69 (–1.604, 0.232)	3	–0.305 (–0.902, 0.294)	3	2.787 (0.926, 4.612)*	0	3.316 (0.573, 5.984)*	0
Heze	0.279 (–0.203, 0.764)	1	0.357 (0.022, 0.694)*	3	2.558 (1.186, 3.948)*	2	1.534 (–0.301, 3.402)	3
Jinan	0.501 (0.003, 1.018)*	4	0.332 (0.008, 0.658)*	4	1.405 (0.203, 2.621)*	3	1.583 (–0.025, 3.218)	0
Jining	0.729 (0.232, 1.229)*	2	0.605 (0.285, 0.925)*	2	1.713 (0.734, 2.701)*	4	1.164 (–0.309, 2.659)	4
Laiwu	0.978 (0.105, 1.858)*	3	1.099 (0.458, 1.743)*	3	1.530 (–0.132, 3.219)	2	2.233 (–0.427, 4.965)	2
Liaocheng	0.388 (–0.053, 0.830)	0	0.262 (–0.052, 0.576)	0	1.217 (0.036, 2.485)*	3	1.697 (0.247, 3.126)*	5
Linyi	0.501 (0.081, 0.922)*	1	0.372 (0.099, 0.646)*	1	2.276 (1.234, 3.328)*	2	0.599 (–0.511, 1.721)	2
Qingdao	0.536 (–0.111, 1.188)	0	0.388 (–0.064, 0.843)	0	3.164 (1.302, 5.059)*	0	2.824 (1.004, 4.677)*	0
Rizhao	0.635 (–0.179, 1.455)	2	0.510 (–0.065, 1.088)	3	1.916 (–0.772, 4.677)	4	1.187 (–1.447, 3.890)	0
Taian	0.561 (0.004, 1.140)*	2	0.314 (0.018, 0.678)*	3	1.490 (0.167, 2.830)*	2	–1.501 (–3.191, 0.218)	5
Weifang	0.448 (0.007, 0.890)*	2	0.406 (0.109, 0.704)*	5	0.656 (–0.156, 1.475)	2	1.245 (–0.052, 2.559)	2
Weihai	–0.667 (–1.907, 0.590)	0	0.254 (–0.361, 0.873)	2	–1.717 (–5.102, 1.789)	0	–1.756 (–4.111, 0.657)	2
Yantai	0.658 (–0.120, 1.442)	5	0.470 (–0.069, 1.012)	2	1.768 (–0.357, 3.938)	2	0.765 (–0.948, 2.508)	2
Zaozhuang	0.904 (0.256, 1.555)*	1	0.542 (0.103, 0.983)*	1	1.484 (0.015, 2.973)*	5	3.013 (0.596, 5.488)*	6
Zibo	0.480 (–0.102, 1.066)	3	–0.189 (–0.567, 0.190)	4	1.210 (0.375, 2.052)*	3	1.602 (0.130, 3.053)*	6
